# High Expression of TIM 3 and Galectin 9 on Immunohistochemistry Staining of Tumor Specimen at Diagnosis in Pediatric Patients with Ewing Sarcoma

**DOI:** 10.33696/cancerimmunol.3.053

**Published:** 2021

**Authors:** Stephanie J. Si, Gerald B. Wertheim, David M. Barrett

**Affiliations:** 1 Children’s Hospital of Philadelphia, Division of Oncology and Center for Childhood Cancer Research, Philadelphia, PA, United States; 2 Children’s Hospital of Philadelphia, Department of Pathology and Laboratory Medicine, Philadelphia, PA, United States; 3 University of Pennsylvania Perelman School of Medicine, Department of Pediatrics, Philadelphia, PA, United States

## Abstract

Significant progress has been made in the advancement of immune system modulation for cancer treatment in recent years. In particular, immune checkpoint inhibitors and chimeric antigen receptor (CAR) T-cell therapy have demonstrated remarkable clinical benefit in relapsed/refractory cancers. However, our understanding of the immuno-oncologic landscape in pediatric solid tumors remains limited and is a barrier to continued progress. We examined the immunohistochemical expression of checkpoint receptors PD-1, TIM-3, LAG-3 and their respective ligands in various pediatric cancers at diagnosis and found high expression of TIM-3/Galectin-9 in the infiltrating cells of Ewing sarcoma. Location of checkpoint receptor/ligand expressions is important, as some staining patterns were only seen along tumor borders. Finally, peripheral T cell function varied significantly among different tumors supporting a complex relationship between the tumor microenvironment and the global immune system.

## Introduction

The concept of immunotherapy has been traced as far back to third century BC Qin dynasty in China through the purposeful inoculation of *Variola minor* to prevent smallpox [1]. Harnessing the knowledge gained through the triumphs of vaccination, use of immune-mediated therapies to treat cancer was first pioneered by William B. Coley, a nineteenth century surgeon at the Hospital for Ruptured and Crippled (now the Hospital for Special Surgery), who noticed that concomitant infection with erysipelas seemed to cause the remission of metastatic sarcoma [2]. Over the next few decades, continued understanding in immunobiology and technologic advancements have led to the exciting development of genetically engineered T cells and immune checkpoint inhibitors that is revolutionizing the treatment of childhood cancers [3,4].

Of particular interest are the recent advances using immune checkpoint blockade as a tool to harness the power of the immune system to fight cancer [5]. When T cells are under constant exposure to cancer cells, they become exhausted and are increasingly less apt in clearing the antigen [6]. Exhausted T cells are unique in that they have a progressive and hierarchical loss of effector functions, altered expression of key transcription factors, and specific metabolic derangements. Furthermore, they also express multiple inhibitor receptors such as Programmed death receptor (PD-1), Lymphocyte activating gene 3 (LAG-3), and T cell immunoglobulin and mucin domain containing 3 (Tim-3) [7]. Targeting of these checkpoint receptors within the tumor microenvironment serves as the basis for the development of drugs such as Nivolumab, a PD-1 inhibitor, that can effectively reverse T cell’s dysfunctional state mediated by the PD-1 pathway and therefore reinvigorate immune responses.

In adult cancer studies, expression of checkpoint receptors and their respective ligands have been demonstrated on immunohistochemistry staining across various tumors. Interestingly, the density, location, and cell types that express these receptors and ligands vary between and within tumor types thus contributing to varying clinical responses to checkpoint inhibitors [8–13]. Nonetheless, Ipilimumab (targeting Cytotoxic T-lymphocyte-associated protein 4 (CTLA-4)) showed an impressive improvement in overall survival in adult patients with advanced melanoma and became the first FDA approved immune checkpoint inhibitor in 2010 [14]. Since then, hundreds of phase I/II trials are underway to evaluate the efficacy of multiple immune checkpoint inhibitor monotherapy or in combination [13].

Owing to the success of checkpoint inhibitors in adult clinical trials, there are now 2 checkpoint inhibitors Ipilimumab and Pembrolizumab that have been approved by the United States Food and Drug Administration (FDA) for pediatric patients with advanced melanoma and relapsed/refractory classical Hodgkin lymphoma respectively [13,15,16]. Much of the pediatrics studies to date have focused on only Programmed death receptor ligand 1 (PD-L1) and PD-1 expression and results have been mixed. Reported PD-L1 expression in at least 1% of tumor cells varies from 9% to 33%, and most samples were from recurrent or metastatic disease [17–22]. Furthermore, LAG-3 and Tim-3 have both gained substantial interest in adult cancer therapy leading to multiple anti-LAG-3 and anti-Tim-3 clinical trials (NCT01968109, NCT02817633). However, the availability of such clinical trials has not yet progressed to pediatric patients as the preclinical data on the expression of these checkpoint receptors on pediatric tumors remains limited.

As such, there is a great need for better understanding of the onco-immunologic landscape of pediatric tumors in order to provide more targeted therapeutic strategies. In this study, we characterized the expression of PD-1, LAG-3, Tim-3, and their respective ligands PD-L1, major histocompatibility complex class II (MHC class II), Galectin-9 in children with Ewing sarcoma and osteosarcoma at diagnosis. We also assessed the interplay between tumor and the effects on circulating T cells to determine the global severity of T cell dysfunction. Examination of the immune-oncologic landscape of these tumor types will shed insight upon potential checkpoint inhibitor therapy, as well as peripheral T cell biomarkers for cellular therapy, as the number of checkpoint receptors simultaneously expressed can be reflective of the severity of T cell dysfunction [6,23].

## Materials and Methods

### Patient selection and clinical protocol

Twenty-five patients with newly diagnosed malignancies were enrolled as part of a clinical trial under an approved Children’s Hospital of Philadelphia Institutional Review Board protocol 12–009915, with written informed consent obtained in accordance with the U.S. common rule (Table 1). Patient age at time of enrollment ranged from 5 years to 22 years of age. Disease groups included Ewing sarcoma (n=17), and osteosarcoma (n=8). Biopsy specimens were obtained at diagnosis (prior to chemotherapy or radiation) via either open biopsy or core-needle biopsy based on surgeon preference. Of all biopsy specimens, 7 of 17 Ewing sarcoma samples and 0 of 10 osteosarcoma samples displayed a definite tumor border as defined by juxtaposition of normal tissue and tumor.

### IHC staining

**Figure 1:**
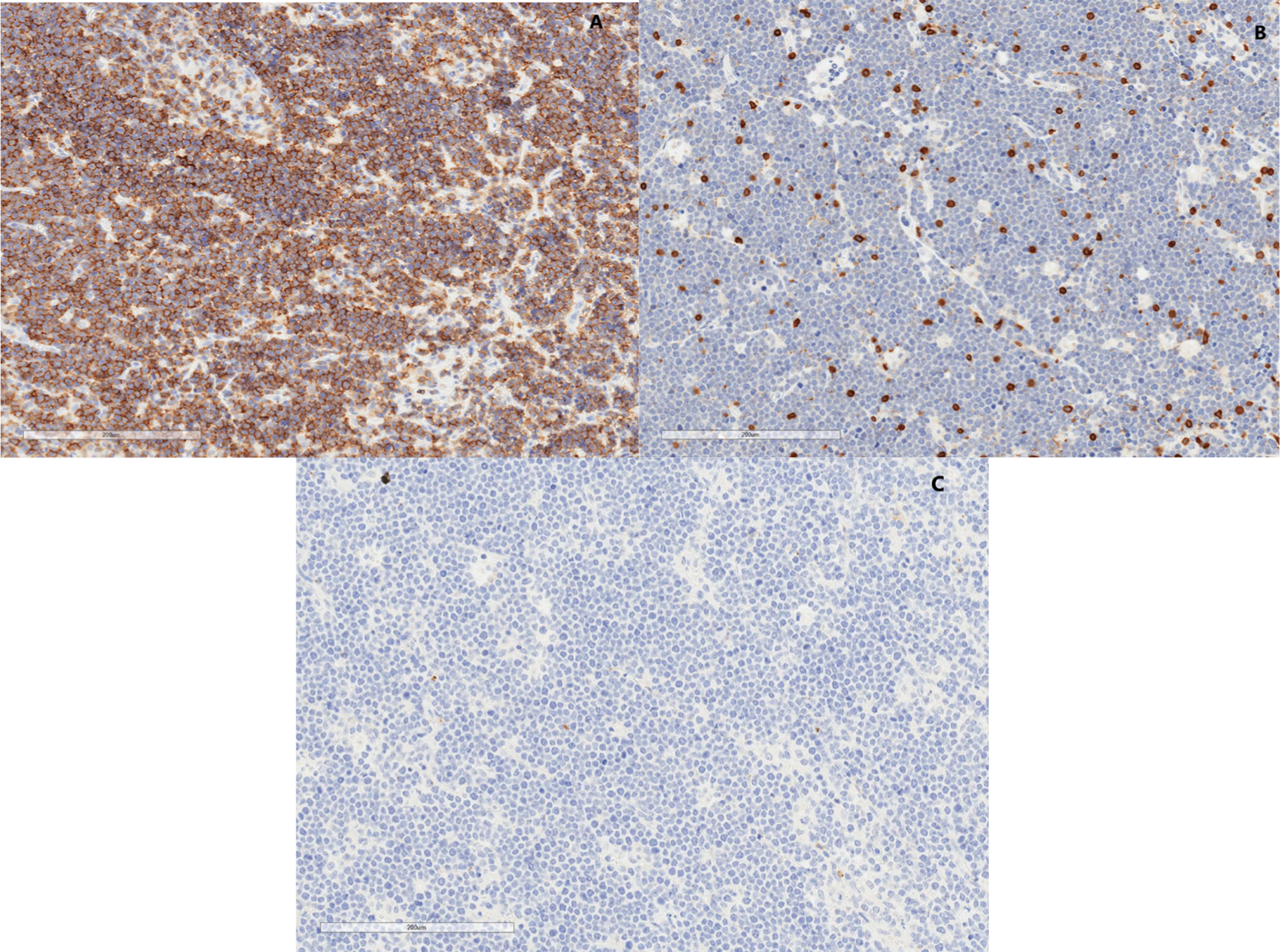
Examples of (A) positive (B) partial positive (C) negative staining.

Formalin fixed paraffin embedded tissue sections were stained with antibodies detailed in (Table 2). Slides were stained with a Leica Bond autostainer (Leica Biosystems) using the Refine staining kit (DS9800). The standard protocol F was followed with the exception of the primary antibody incubation time which was extended to 60 minutes. For fluorescent staining (ALK, Galectin 9), slides were processed by hand using standard methods. Briefly, slides were deparaffinized in xylene and rehydrated in a descending series of ethanol. Antigen retrieval was performed using a pressure cooker (Biocare Medical) with antigen unmasking solution (Vector Labs). Blocking was performed in 0.5% SDS for 20 minutes, followed by 2% fetal bovine serum for 20 minutes. Primary antibodies were incubated at room temperature for 60 minutes followed by appropriate Alexa fluor 488 or 594 Secondary antibody incubation (Invitrogen) at a 1:200 dilution for 1 hour at room temperature. Slides were counter stained with DAPI (Sigma) and scanned at 20x magnification using an Aperio CS-O (brightfield) or fluorescent scanner. Results of IHC staining pattern were reviewed with a single pathologist and graded as “negative”, “rare” or “positive” (Figure 1).

### Flow cytometry

Flow cytometry was performed on peripheral blood T cells with the following antibodies: PD1-BV510 (BD Biosciences, #563076), Tim3-BV421 (BD Biosciences, #565562), and LAG3-PerCP-eFluor 710 (BD Biosciences, #3DS223H). Samples were washed twice and flow cytometry was conducted with FACS Verse (BD Biosciences). Due to lack of available samples, only seven peripheral blood samples were analyzed from the osteosarcoma group and 8 peripheral blood samples were analyzed from the Ewing sarcoma group.

### *Ex vivo* T cell expansion and culture

Lymphocytes were harvested from peripheral blood and expanded using CD3/CD28 beads as previously described [24]. After 7 days, beads were magnetically removed and cells were cultured for an additional seven days. Fold expansion was calculated by dividing maximum cell count by the starting cell count.

### Statistical analysis

Flow cytometry data were analyzed using FlowJo software (v10.6.1; Tree Star). Subsequent statistical analysis was performed using Prism 8 (Version 8.2.0, Graphpad Software, La Jolla, CA) using analysis of variance (ANOVA) testing for group comparisons. Significant results are denoted by p-value <0.05.

### Gene expression analysis

RNA was isolated using the RNeasy mini plus kit (QIAGEN) and hybridized to the nCounter CAR-T characterization panel (human codeset). RCC files containing raw counts for 770 genes provided from NanoString were loaded into nSolver Analysis Software 3.0 and normalized for housekeeping genes and positive controls. Sequence data was analyzed using Morpheus (Broad Institute) to map transcripts and calculate reads per kilobase million (SW780 cDNA expression) or transcripts per million (UM-UC-9).

## Results

### Negative checkpoint receptors (NCR) and ligands expression pattern in pediatric tumors vary based upon tumor type and location within the tumor

Eighteen biopsy samples were stained with PD-1, PD-L1, TIM-3, Galectin-9, LAG-3, and MHC Class II and underwent pathology review. Tumor infiltrating lymphocytes (TILs) were differentiated against tumor cells based on expression of CD3, CD20, or CD68 on IHC staining. For Ewing sarcoma, 4 of 10 (40%) samples had positive PD-1 staining of tumor cells, and 10 of 10 (100%) samples had positive staining for both TIM-3 and Galectin-9 in infiltrating non-tumor cells (Table 3A). To confirm our findings, 7 additional diagnostic Ewing sarcoma biopsy samples demonstrated 7 of 7 (100%) positive staining for TIM-3, and 5 of 7 (71%) positive staining for Galectin-9 (Table 3B). This expression pattern is different from osteosarcoma staining as none of the samples had positive PD-1 staining of tumor cells, and only 5 of 8 (62.5%) osteosarcoma samples stained positively for both TIM-3 and Galectin-9 in non-tumor cells (Table 3C). PD-L1 and LAG-3 staining was not observed in either Ewing sarcoma or osteosarcoma samples. Details of all staining results can be found in Table 4.

**Figure 2:**
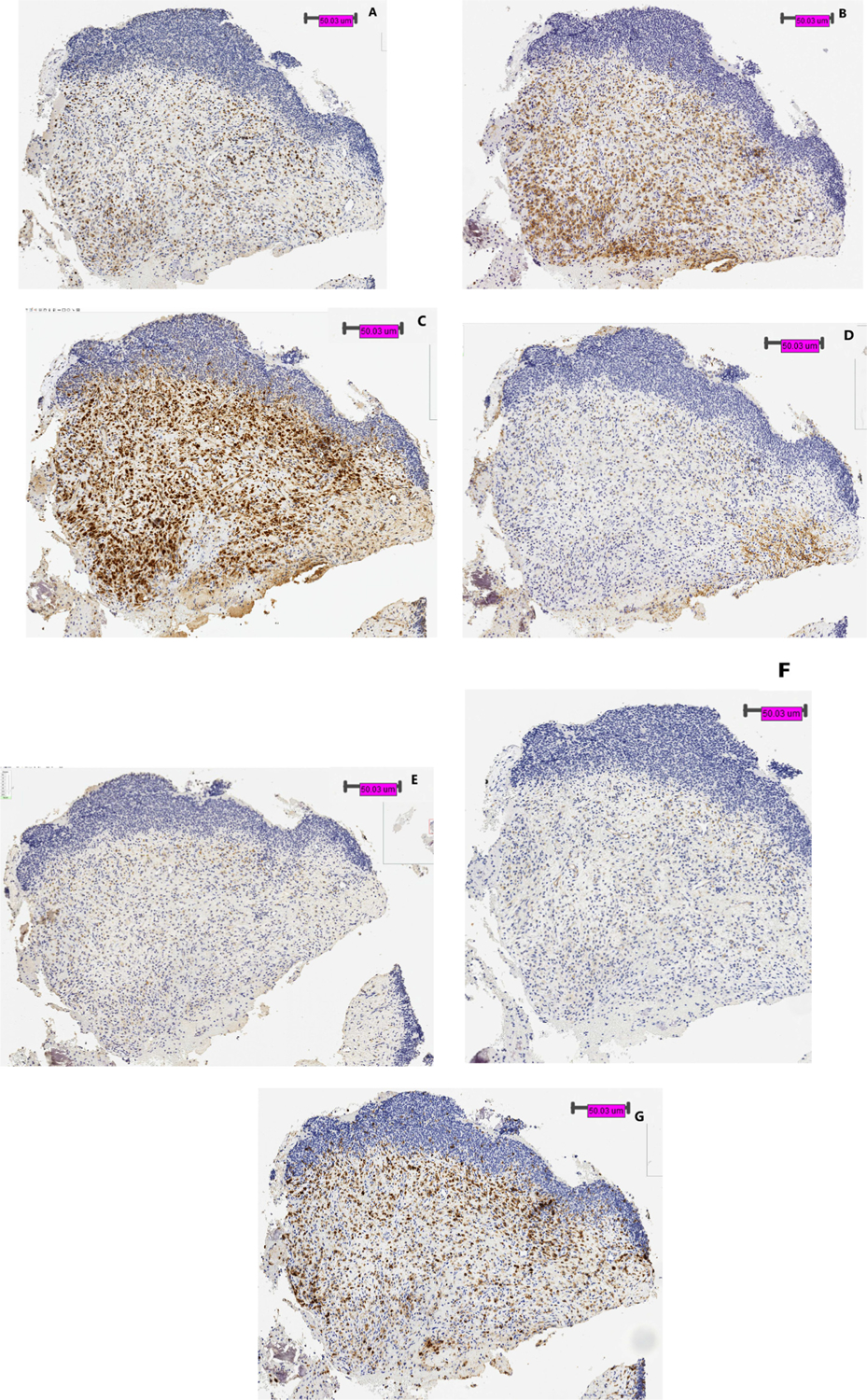
Biopsy specimen of Ewing sarcoma in left femur at diagnosis. Panel **(A)** shows CD3 staining. By IHC staining, **(B)** TIM-3 and **(C)** Galectin-9 are both strongly positive on non-tumor cells only and location of checkpoint receptor/ligand congregating at tumor border, **(D)** PD-1 and **(E)** PD-L1 are partially positive on non-tumor cells only, **(F)** LAG-3 is negative on both tumor and non-tumor cells, and **(G)** MHC class II is positive on tumor cells only.

Aside from the differences in overall expression pattern, location of where NCR and ligands were expressed is also noteworthy. We identified a tumor border in 7 of 17 (41%) Ewing sarcoma samples. As represented in Figure 2, staining of checkpoint receptor and ligands was seen in Ewing sarcoma cells congregating along and outside of the tumor border. Few cells were identified within the tumor itself.

**Figure 3:**
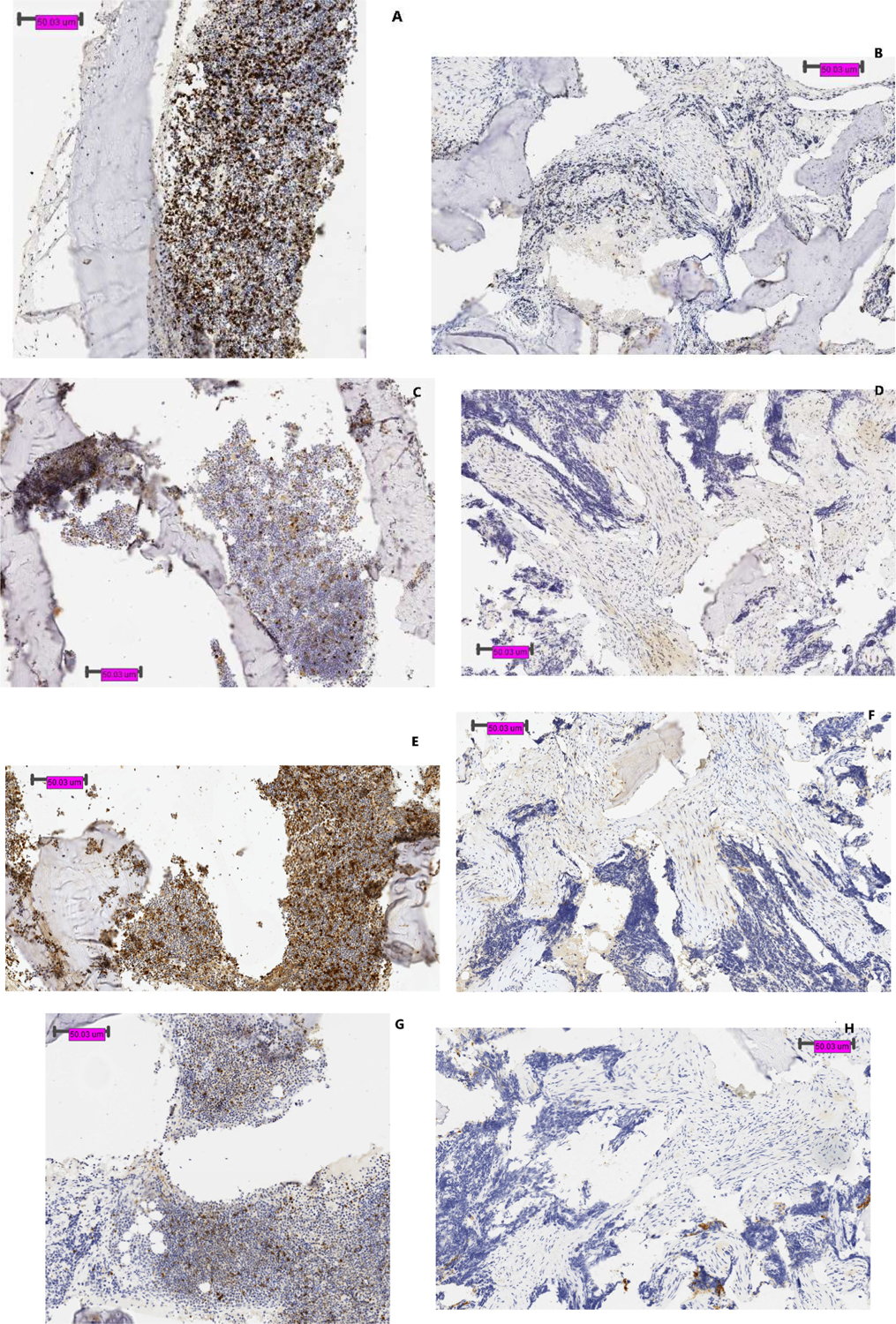

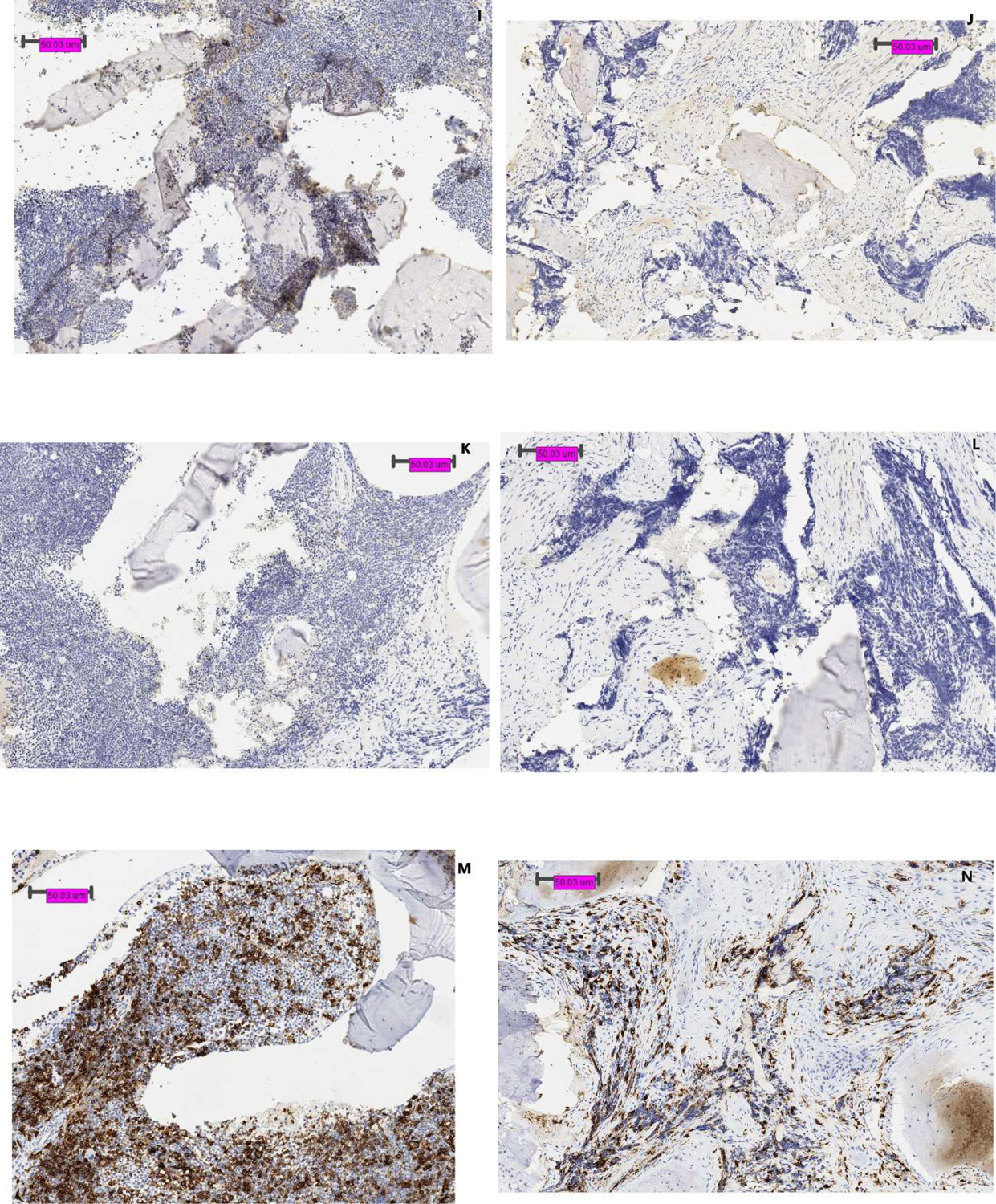
Biopsy specimen of Ewing sarcoma in vertebral column at diagnosis. Shown here is the comparison between staining profile on tumor and bone marrow. Panel **(A)** shows CD3 staining on bone marrow, and **(B)** tumor. Panels **(C, E, G, I, K, M)** and **(D, F, H, J, L, N)** shows TIM-3, Galectin-9, PD-1, PD-L1, LAG-3, and MHC class II on bone marrow and tumor respectively.

In Figure 3, biopsy specimen includes normal bone marrow in a patient with Ewing sarcoma. Whereas PD-1 stained positive in bone marrow, biopsy of Ewing sarcoma was negative for PD-1 in non-tumor cells. Remainder of staining pattern between tumor and normal tissue appeared to correlate well with each other. Additional staining expression of CD20, 56, and 68 is included in Supplemental data.

### No correlation is found between expression of NCR and ligands on tumor and peripheral T cells

**Figure 4:**
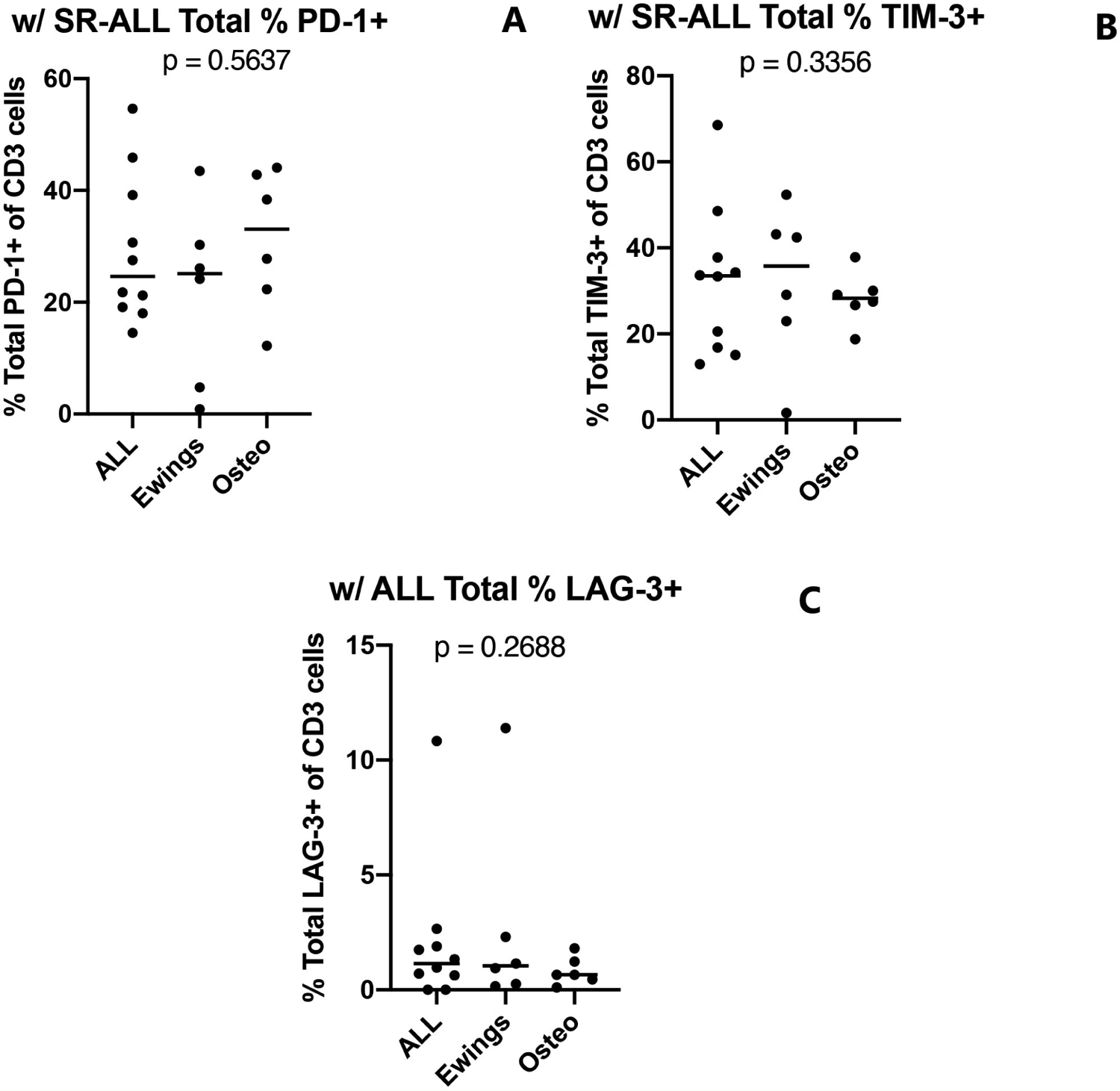
No differences seen in overall NCR expression on peripheral T cells across tumor types. Percentage of CD3^+^ cells expressing (A) PD-1 (B) TIM-3, and (C) LAG-3. Bars represent the median. P values calculated using a Mann-Whitney test.

Using standard risk B-cell acute lymphoblastic leukemia (SR B-ALL) as baseline, we examined the expression of receptor and ligands on circulating T cells between the two disease groups and found no statistically significant difference between overall expression of PD-1, TIM-3, or LAG-3 (Figure 4). The reason for choosing SR B-ALL samples as control is due to previously published data that confirmed close resemblance of peripheral T cell subset distribution and biology to healthy children [24–26], as we do not have access to blood from healthy children under these study protocols.

We then compared the expression of NCR and ligands on peripheral T cells to the expression pattern on tumor samples, and no definitive correlation can be made. Whereas TIM-3 and Galectin-9 were highly expressed on non-tumor cells in all Ewing sarcoma biopsies, an increase in TIM-3 expression on peripheral T cells was not found when compared to the osteosarcoma group. Similarly, a lack of PD-1 expression on osteosarcoma tissue samples did not correlate with low expression of PD-1 on peripheral T cells.

### T cell expansion potential differs between disease groups at diagnosis and is correlated with total number of NCRs expressed on peripheral T cells

**Figure 5:**
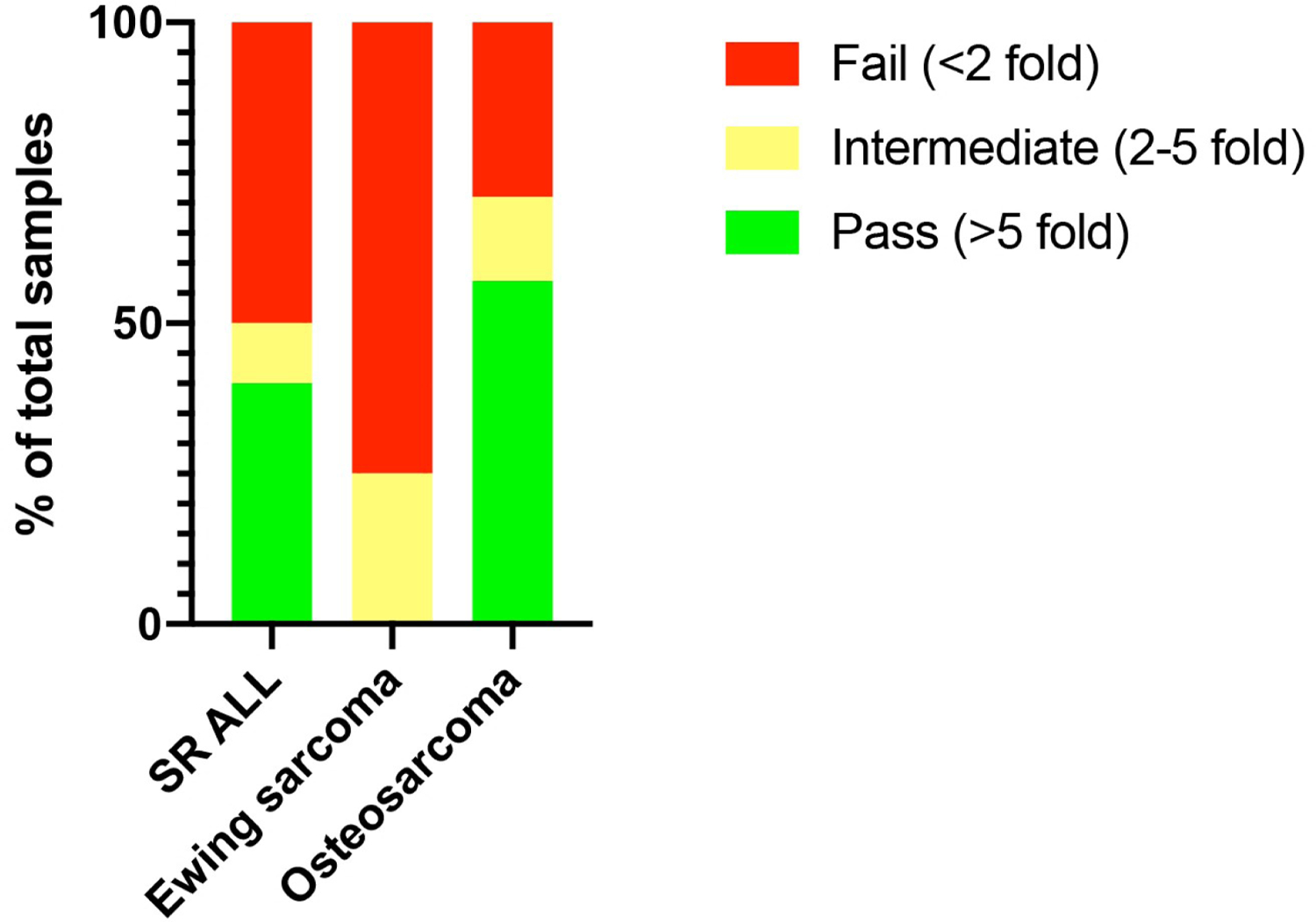
Percentage of peripheral blood T cells at diagnosis that demonstrated >5 fold *in vitro* expansion.

We previously reported a threshold of >5-fold expansion during stimulation with CD3/28 beads was associated with a high likelihood of successful clinical expansion [24]. Therefore, we applied the same threshold to stratify this analysis into samples that “passed” expansion (>5 fold), “failed” expansion (<2 fold), and “indeterminate” (2–5 fold). In this study, SR ALL patients had a higher initial pass rate at diagnosis (40%) than patients with Ewing sarcoma (0%), which is consistent with our previous findings [24]. In contrast, osteosarcoma had a higher initial pass rate than SR-ALL at diagnosis (57%). This is correlated with osteosarcoma patients having the lowest percentage of peripheral T cells that had simultaneous expression of all three NCRs (0.38%), compared to SR ALL (0.7%) and Ewing sarcoma (1.61%) (Table 5, Figure 5).

## Discussion

In this study, we characterized the expression of PD-1, PD-L1, TIM-3, Galectin 9, LAG-3, MHC Class II on diagnostic tissue samples from patients with Ewing sarcoma and osteosarcoma. Although we observed significant variability in the expression pattern between tumor types, two interesting findings were observed. First, both TIM-3 and Galectin 9 were highly expressed on non-tumor cells in Ewing sarcoma samples at diagnosis. This finding is significant as it can potentially serve as a platform for clinical trials using anti-TIM-3 and anti-Galectin-9 monoclonal antibody in select pediatric solid tumors. However, as few studies to date have evaluated the expression of checkpoint receptors and ligands other than PD-1 and PD-L1, a larger sample size is needed to confirm this finding [27]. Second, osteosarcoma tumor cells at diagnosis did not express any checkpoint receptors and ligands except Galectin-9, and non-tumor cells were also negative for PD-1, PD-L1, and LAG-3, suggesting that these tumors may be resistant to currently available checkpoint inhibitors if used at diagnosis.

The lack of checkpoint receptors and ligands expression on both tumor and non-tumor cells in osteosarcoma diagnostic samples is unique compared to both Ewing sarcoma. In Ewing sarcoma samples, only PD-L1, LAG-3, and MHC class II were negative on tumor cells, and LAG-3 was also negative on non-tumor cells. Not only does this highlight the variability of the immune-oncologic landscape between different tumor types at diagnosis, it also suggests that up front therapy with checkpoint blockade may not be an ideal choice of therapy for every tumor type.

Despite our findings, it is important to note that the staining pattern observed in our study is different from previously published studies. Factors that contribute to this difference includes the timepoint of which biopsy specimens were obtained: at diagnosis, during treatment, post treatment, or at disease recurrence. In osteosarcoma, expression of checkpoint receptors has been shown to vary extensively [28–31]. Shen et al. found a significant range of PD-L1 expression in osteosarcoma specimens, with 6 of 38 (15.8%) samples having negative expression, 4 of 38 (10.5%) samples with low PD-L1 expression, 19 of 38 (38%) with intermediate PD-L1 expression, and 9 of 38 (23.7%) samples with high PD-L1 expression [30]. However, specimens in this study include diagnostic, treated, and recurrent tumors. Likewise, Chen et al. confirmed that variations in specimen collection time points significantly alter landscape of checkpoint receptors and ligands expression, as PD-1 and PD-L1 were uniformly absent in post-treatment specimens even when its expression were present at diagnosis and in recurrent tumors within the same patient [29]. However, interestingly, even for samples that were all obtained at diagnosis, variations in checkpoint receptors and ligands expression can still be seen. Chen et al. reported PD-1 positivity in 8 of 15 (53%) osteosarcoma samples at diagnosis [29], while Alves et al. noted only “rare” PD-1 positivity in 7 of 21 (33.3%) in diagnostic biopsies of osteosarcoma, with the remainder of the samples having negative expression of PD-1 (14 of 21, 66.6%) [28]. It is important to note that there is no unified immunostaining scoring system between the above studies. Criteria for “positive” and “rare” expression of checkpoint inhibitors varied, with some studies defining “positive” as ≥50% of tumor cells analyzed at 20x magnification, while other studies relied on pathologists’ subjective interpretation of “weak”, “moderate” and “strong” staining patterns [20,29]. The significant variations in expression pattern of checkpoint receptors and ligands across different tumor types points to the importance of a comprehensive investigation of immunohistochemistry staining with uniformity of staining procedures and analysis criteria, while paying close attention to the timepoints from which the specimens were obtained from.

Another important factor contributing to the variation in immunohistochemistry staining pattern lies in the location of where the biopsy was obtained in the tumor. As evidenced by the Ewing sarcoma biopsy sample in Figure 2, non-tumor cells expressing various checkpoint receptors and ligands are congregated at the tumor border, and not dispersed within the tumor. Several other adult studies have described similar findings. Taube et al. demonstrated geographic restriction of PD-L1 and LAG-3 positive tumor cells and non-tumor cells at the tumor border of metastatic melanoma [32]. Aust et al. also reported PD-L1 expression in recurrent serous ovarian tumor was limited to tumor cells at the tumor-stroma border [33]. However, owing to the different biopsy techniques and tumor locations, biopsies that include tumor borders are not often feasible. Of the 25 biopsy samples included in this study, only 7 samples included a tumor border. As a result, expression of checkpoint receptors and ligands may be under reported in samples that did not include a tumor border.

Next, we sought to evaluate the relationship between the expression of checkpoint receptors on peripheral T cells and pediatric tumor biopsy samples at diagnosis. Although expression of TIM-3 and Galectin 9 were seen on most Ewing sarcoma diagnostic biopsy samples, expression of TIM-3 on peripheral T cells at diagnosis was similar to other tumor types. Similarly, although expression of PD-1 and PD-L1 were negative across all osteosarcoma diagnostic biopsy samples, expression of PD-1 on peripheral T cells at diagnosis again did not differ from other tumor types. Our data shows that peripheral blood T cells do not reflect the tumor environment at diagnosis, therefore potentially limiting this parameter’s prognostic value. However, it is interesting to note that a recent study did find a correlation between expression of select checkpoint receptors and TILs. Li et al. looked at a total of 131 adult patients with newly diagnosed breast, kidney, lung, liver, cervical, esophageal, gastric, and colorectal cancer and found that the frequencies of PD-1 and TIM-3 expression in peripheral T cells positively correlated with their respective frequencies in TILs. Other checkpoint receptors such as B and T lymphocyte attenuator (BTLA) and T cell immunoreceptor with Ig and ITIM domains (TIGIT) were not shown to have statistically significant correlation between peripheral T cells and TILs [34]. Although the difference in our results may be a reflection of the limited number of patients in our study, it is also possible that different tumors impart a different effect on peripheral T cells as the tumors evaluated in Li et al. did not include Ewing sarcoma, osteosarcoma, or NHL samples. Furthermore, pediatric tumors may have unknown intrinsic biologic differences compared to adult tumors (i.e. carcinomas) which contribute to varying onco-immunologic landscapes, highlighting the importance of future similar studies in order to build upon our current understanding of this intricate relationship.

**Figure 6:**
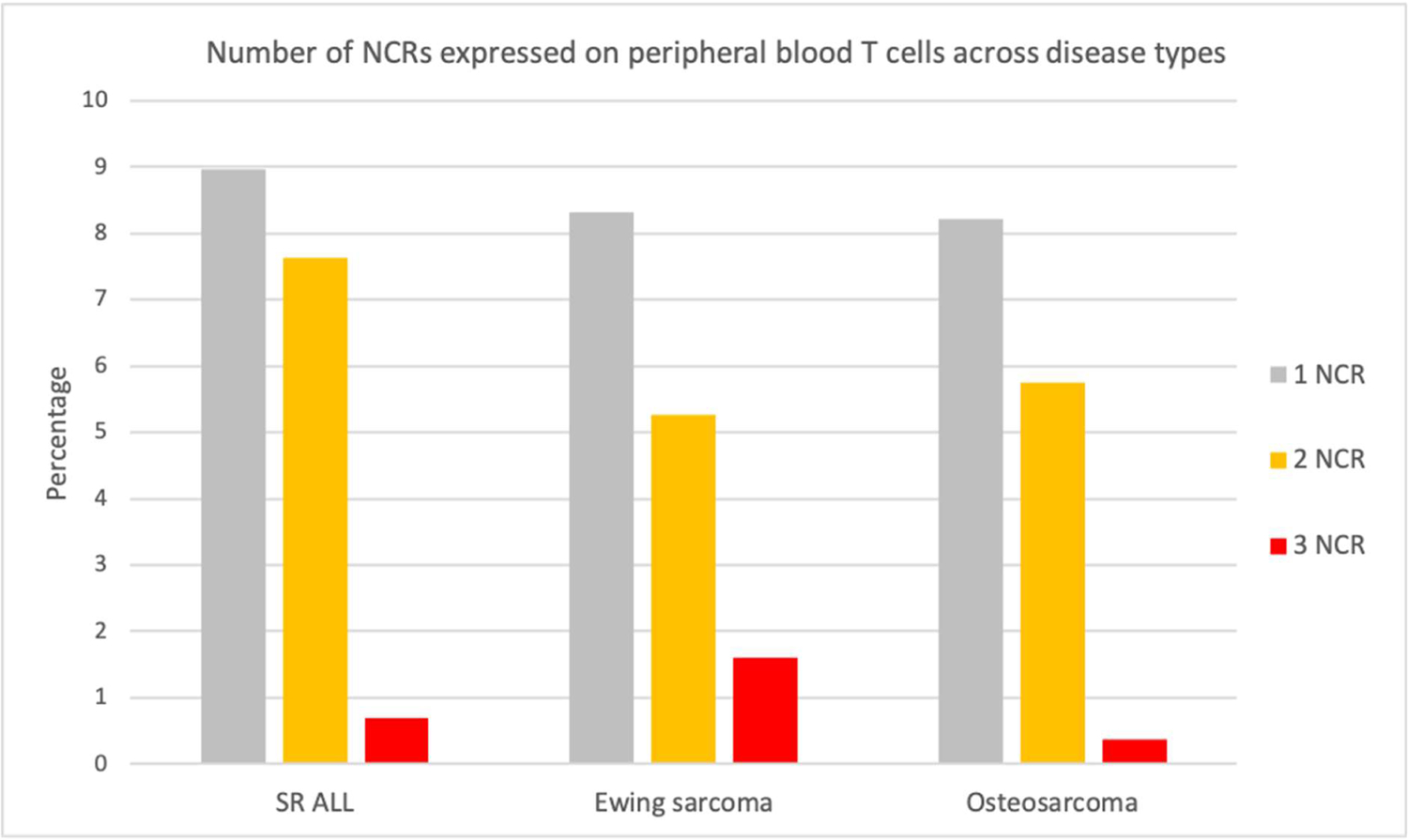
Percentage of peripheral blood T cells at diagnosis with simultaneous expression of 1, 2, or all 3 NCRs PD-1, TIM-3, and LAG-3.

Although the expression profile of checkpoint receptors appear to be similar across the tumor types, qualitative differences were observed through differences *in vivo* expansion potential. Using a threshold of >5-fold T cell expansion during stimulation with CD3/CD28 beads as a marker for “healthy” effector function, we demonstrate that 57% of osteosarcoma samples in this study had >5-fold T cell expansion, compared to 0% of Ewing sarcoma (Figure 6). This finding reassuringly confirms our prior work that demonstrates a higher pass rate in diagnostic osteosarcoma than Ewing sarcoma samples [24]. This may in part be due to the distribution of peripheral T cell subsets with osteosarcoma patients having more naïve and stem central memory T cells than Ewing sarcoma patients [24], as well as a relative preservation of T cell effector function as only 0.38% of total peripheral T cells in osteosarcoma samples had co-expression of all three checkpoint receptors at diagnosis (Table 5, Figure 5). This finding again supports that different tumor microenvironments may be associated with global immune changes by varying degrees and mechanisms.

Limitations of this study includes small sample size within each tumor group which may contribute to the variations in IHC staining results seen in our study compared with prior studies. IHC staining results may also be affected by the decalcification process of bone tumors, as well as crush artifacts in biopsy specimens. We are at risk of observational bias by working with only one pathologist, and interpretation subjectivity remains to be a barrier that is difficult to overcome. Use of different antibodies against checkpoint receptors and ligands, along with different staining techniques further complicate results. Sampedro-Nunez et al. reported the use of three different antibodies against PD-L1 but only one antibody stained positive for PD-L1 in their tumor samples [35]. Finally, the lack of biopsy samples that included a tumor border may also underestimate the true incidence of checkpoint receptors and ligands expression found within and around the tumor.

In summary, we provide the first ever study that describe the comprehensive onco-immunologic landscape in pediatric solid tumors by correlating expression of checkpoint receptor and ligand expression on treatment naïve biopsy samples with peripheral blood T cells. We demonstrate high expression of TIM-3 and Galectin 9 positivity on immunohistochemistry staining in Ewing sarcoma, along with a relative lack of checkpoint receptor and ligand expression in osteosarcoma samples. However, such findings on biopsy samples are not reflected in peripheral blood T cells therefore suggesting a more complex interaction between the tumor microenvironment and the global immune system as well as highlighting the limitations of peripheral blood biomarkers in this setting. Further understanding is urgently needed in order to maximize the clinical benefit of checkpoint inhibitors in pediatric solid tumors.

## Supplementary Material

JCAI-21-054_Supplementary file

## Figures and Tables

**Table 1:** Summary of patient characteristics including tumor types, and number of diagnostic biopsy samples that included a tumor border.

Disease	Age (years)	Number of patients	Biopsies with tumor border
Ewing sarcoma	12 ± 5	10	6 of 10
NHL	18 ± 4.7	8	3 of 8
Osteosarcoma	16 ± 4.6	8	0 of 10

**Table 2:** Summary of antibodies used in immunohistochemistry staining protocols.

Antibody	Clone	Vendor	Catalog Number	Dilution	Antigen Retrieval	Protocol
CD20	L26	Dako	M0755	1:1K	E2-20	Leica Bond Refine
CD68	Kp1	Dako	M0814	1:4K	E2-20	Leica Bond Refine
PD1	EH33	Cell Signaling	43248	1:100	E2-20	Leica Bond Refine
Tim-3	D5DR	Cell Signaling	45208	1:50	E2-20	Leica Bond Refine
Lag-3	17B4	LSBio	C18692	1:200	E1-20	Leica Bond Refine
PD-L1	E13N(R)	Cell Signaling	13684	1:200	E1-20	Leica Bond Refine
CD3	n/a	Dako	A0452	1:100	E1-20	Leica Bond Refine
CD56	VRQ-42	Cell Marque	156-R-95	1:400	E2-20	Leica Bond Refine
ALK	D5F3	Cell Signaling	3633	1:200	P.C. Citrate	IF
Galectin 9	n/a	R&D Systems	AF2045	1:50	P.C. Citrate	IF
MHCII HLA-DR	TAL.1B5	Dako	M0746	1:100	E1-20	Leica Bond Refine

**Table 3:** High expression of TIM-3 and Galectin-9 staining in (A) Ewing sarcoma, which is further confirmed in 7 additional diagnostic biopsy samples (B). This is in stark contrast to the staining pattern seen in Osteosarcoma (C).

**A**	**Ewing (n=10)**							
**PD-1**			**TIM-3**			**LAG-3**		
+	+/−	−	+	+/−	−	+	+/−	−
1/10	3/10	6/10	10/10	0/10	0/10	0/10	0/10	10/10
10%	30%	60%	100%	0%	0%	0%	0%	100%
**PD-L1**			**Galectin 9**			**MHC Class II**		
+	+/−	−	+	+/−	−	+	+/−	−
0/10	1/10	9/10	10/10	0/10	0/10	10/10	0/10	0/10
0%	10%	90%	100%	0%	0%	100%	0%	0%
**B**	**Ewing (n=17)**							
**TIM-3**								
+	+/−	−						
17/17	0/17	0/17						
100%	0%	0%						
	**Galectin 9**							
+	+/−	−						
15/17	0/17	2/17						
88.2%	0%	11.8%						
**C**	**Osteosarcoma n=8**							
**PD-1**			**TIM-3**			**LAG-3**		
+	+/−	−	+	+/−	−	+	+/−	−
0/8	1/8	7/8	5/8	1/8	2/8	0/8	0/8	8/8
0%	12.5%	87.5%	62.5%	12.5%	25%	0%	0%	100%
**PD-L1**			**Galectin 9**			**MHC Class II**		
+	+/−	−	+	+/−	−	+	+/−	−
0/8	0/8	8/8	5/8	0/8	3/8	8/8	0/8	0/8
0%	0%	100%	62.5%	0%	37.5%	100%	0%	0%

**Table 4:** Summary of all staining results for Ewing sarcoma, Osteosarcoma, and NHL.

**Ewing (n=10)**								
**PD-1**			**TIM-3**			**LAG-3**		
+	+/−	−	+	+/−	−	+	+/−	−
1/10	3/10	6/10	10/10	0/10	0/10	0/10	0/10	10/10
10%	30%	60%	100%	0%	0%	0%	0%	100%
**PD-L1**			**Galectin 9**			**MHC Class II**		
+	+/−	−	+	+/−	−	+	+/−	−
0/10	1/10	9/10	10/10	0/10	0/10	10/10	0/10	0/10
0%	10%	90%	100%	0%	0%	100%	0%	0%
**Osteosarcoma n=8**								
**PD-1**			**TIM-3**			**LAG-3**		
+	+/−	−	+	+/−	−	+	+/−	−
0/8	1/8	7/8	5/8	1/8	2/8	0/8	0/8	8/8
0%	12.5%	87.5%	62.5%	12.5%	25%	0%	0%	100%
**PD-L1**			**Galectin 9**			**MHC Class II**		
+	+/−	−	+	+/−	−	+	+/−	−
0/8	0/8	8/8	5/8	0/8	3/8	8/8	0/8	0/8
0%	0%	100%	62.5%	0%	37.5%	100%	0%	0%
**NHL n=8**								
**PD-1**			**TIM-3**			**LAG-3**		
+	+/−	−	+	+/−	−	+	+/−	−
7/8	1/8	0/8	3/8	3/8	2/8	4/8	4/8	0/8
87.5%	12.5%	0%	37.5%	37.5%	25%	50%	50%	0%
**PD-L1**			**Galectin 9**			**MHC Class II**		
+	+/−	−	+	+/−	−	+	+/−	−
3/8	3/8	2/8	6/8	2/8	0/8	4/8	2/8	2/8
37.5%	37.5%	25%	75%	25%	0	50%	25%	25%

**Table 5:** Peripheral T cells at diagnosis that expressed only 1 NCRs, 2 NCRs, and all 3 NCRs in different disease groups.

	1 NCR	2 NCR	3 NCR
SR ALL	8.97%	7.63%	0.7%
Ewing sarcoma	8.31%	5.27%	1.61%
Osteosarcoma	8.21%	5.75%	0.38%

## Abbreviations:

PD-1: Programmed Death receptor
PD-L1: Programmed Death receptor Ligand 1
LAG-3: Lymphocyte Activating Gene 3
TIM-3: T cell Immunoglobulin and Mucin domain containing 3
CTLA-4: Cytotoxic T-Lymphocyte-Associated protein 4
FDA: Food and Drug Administration
MHC: Major Histocompatibility Complex
NHL: Non-Hodgkin Lymphoma
DLBCL: Diffuse Large B Cell Lymphoma
PMBCL: Primary Mediastinal B Cell Lymphoma
ANOVA: Analysis of Variance
SR B-ALL: Standard Risk B-cell Acute Lymphoblastic Leukemia
TILs: Tumor Infiltrating Lymphocytes
BTLA: B and T Lymphocyte Attenuator
JAK/STAT: Janus Activated Kinase/Signal Transducer and Activator of Transcription
TIGIT: T cell Immunoreceptor with Ig and ITIM domains
CAR: Chimeric Antigen Receptor
IFN-γ: Interferon Gamma
AP-1: Activator Protein 1
IRF: Interferon Regulatory Factor
NCR: Negative Checkpoint Receptor
