# Supplementary material for: High Expression of TIM 3 and Galectin 9 on Immunohistochemistry Staining of Tumor Specimen at Diagnosis in Pediatric Patients with Ewing Sarcoma
Source: J Cancer Immunol (Wilmington). Author manuscript; Available in PMC 2022 Aug 4. (PMC9351179; doi:10.33696/cancerimmunol.3.053)
Supplement: JCAI-21-054_Supplementary file [file NIHMS1818222-supplement-JCAI-21-054_Supplementary_file.pdf]

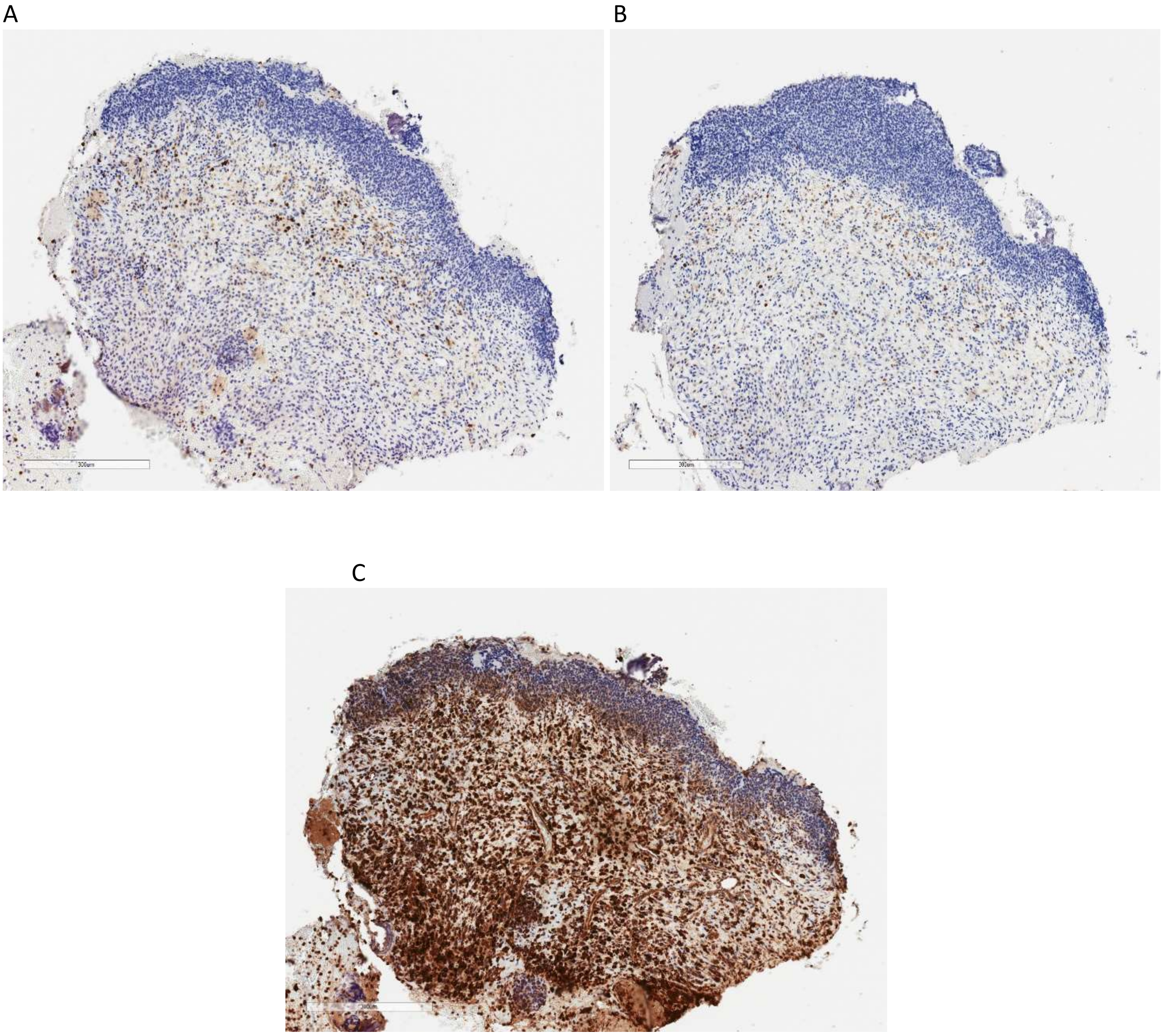

Supplemental Figure 1: Biopsy specimen of Ewing sarcoma in left femur at diagnosis. Panel (A) shows CD20 staining, panel (B) shows CD56 staining, and panel (C) shows CD68 staining.

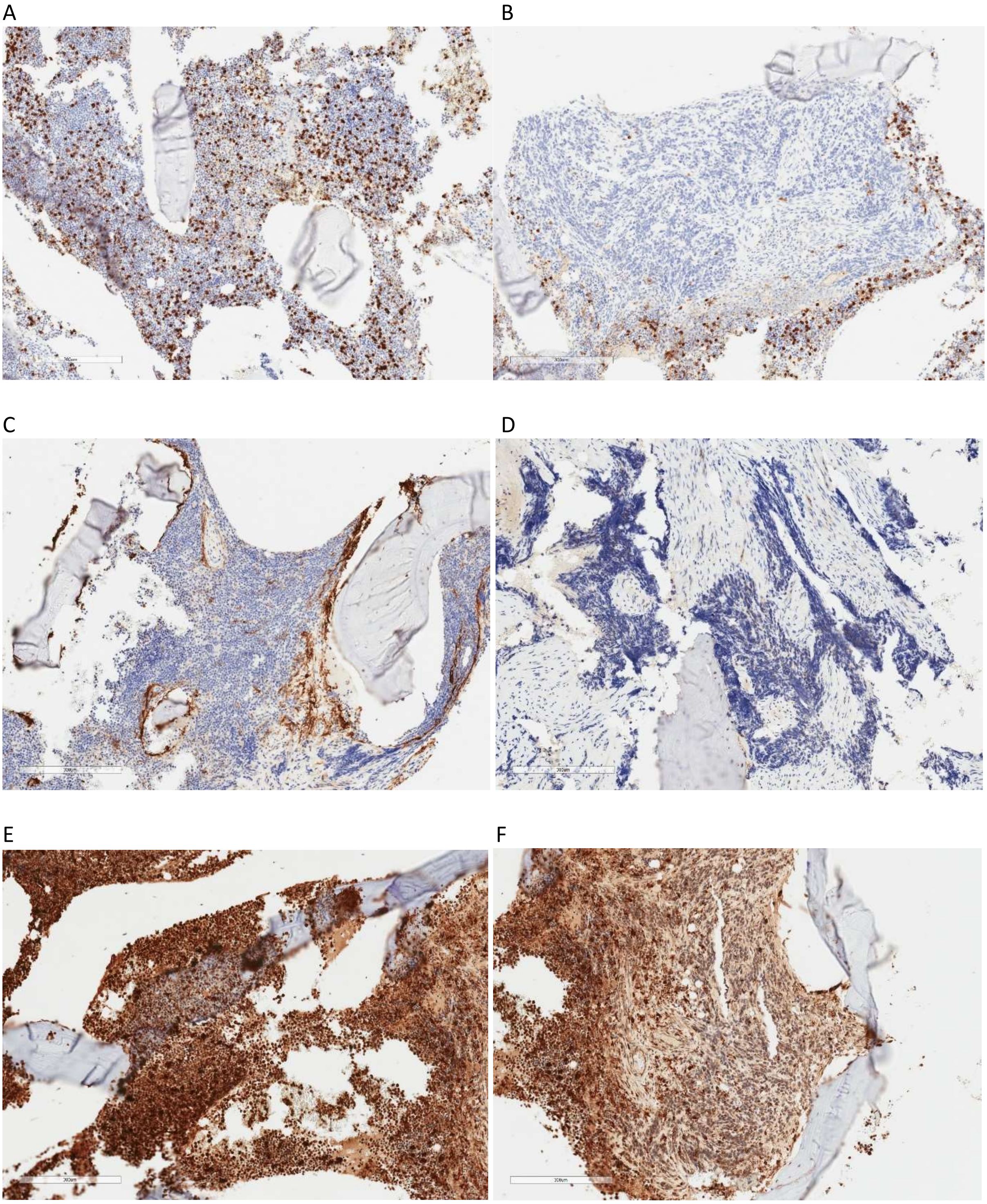

Supplemental Figure 2: Biopsy specimen of Ewing sarcoma in vertebral column at diagnosis. Shown here is the comparison between staining profile on tumor and bone marrow. Panel (A) shows CD20 staining on bone marrow, and (B) tumor. Panels (C, E) and (D, F) shows CD56, and CD68 on bone marrow and tumor respectively.
